# Clinical and Mutational Features of Three Chinese Children with Congenital Generalized Lipodystrophy

**DOI:** 10.4274/jcrpe.3556

**Published:** 2017-03-01

**Authors:** Xueying Su, Ruizhu Lin, Yonglan Huang, Huiying Sheng, Xiaofei Li, Tzer Hwu Ting, Li Liu, Xiuzhen Li

**Affiliations:** 1 Guangzhou Women and Children’s Medical Center, Department of Genetics and Endocrinology, Guangzhou, China; 2 Guangzhou Women and Children’s Medical Center, Division of Medical Imaging, Guangzhou, China; 3 Univeristy Putra Malaysia, Department of Pediatrics, Selangor, Malaysia

**Keywords:** Lipodystrophy, hypertriglyceridemia, Diabetes, Cardiomyopathy

## Abstract

**Objective:**

To investigate the clinical and molecular features of congenital generalized lipodystrophy (CGL) in three Chinese patients with various typical manifestations.

**Methods:**

Data on clinical symptoms, results of laboratory analyses, and previous treatments in three Chinese patients were collected by a retrospective review of medical records. All coding regions and adjacent exon–intron junction regions of *AGPAT2* and *BSCL2* genes were amplified by polymerase chain reaction and sequenced.

**Results:**

Generalized lipodystrophy, acanthosis nigricans, muscular hypertrophy, severe hypertriglyceridemia, and hepatomegaly were features in all three patients. Patient 1 developed diabetes mellitus at the early age of 2 months and he was the youngest CGL patient reported with overt diabetes. Patient 2 was found to have cardiomyopathy when she was aged 6 months. All of the patients were found to have mutations in the *BSCL2* gene, but none of these was a novel mutation. We did not find any AGPAT2 mutation in our patients.

**Conclusion:**

All of our patients exhibited characteristic features of CGL due to mutations in the *BSCL2* gene.

**WHAT IS ALREADY KNOWN ON THIS TOPIC?**

Congenital generalized lipodystrophy (CGL) is a rare autosomal recessive disorder characterized by generalized lipodystrophy, hypertriglyceridemia, hyperinsulinemia, hepatomegaly, and acanthosis nigricans.

**WHAT THIS STUDY ADDS?**

This study revealed the clinical features, molecular characteristics, and treatments of three Chinese CGL patients in order to better understand the diagnosis, clinical treatment, and prognosis of this rare disease.

## INTRODUCTION

Congenital generalized lipodystrophy (CGL), first described by Berardinelli in 1954 (1), is a rare autosomal recessive disorder characterized by near-complete absence of adipose tissue at birth (2,3). Affected individuals have severe hypertriglyceridemia, hyperinsulinemia, hepatomegaly, and often widespread acanthosis nigricans (4). Diabetes mellitus usually appears by the second decade or in adulthood (5,6,7,8). Additionally, hypertrophic cardiomyopathy is a classical late (second or third decade) complication (9,10) which has only been occasionally described in infancy or childhood (11,12,13,14). Currently, the disease is classified into four types according to clinical characteristics and type of mutations. Although four different CGL syndromes have been defined, 95% of reported patients correspond to CGL1 or CGL2 patients. CGL1 is caused by mutations in *AGPAT2*. The *AGPAT2* gene is located on chromosome 9q34 and encodes 1-acylglycerol-3-phosphate O-acyltransferase 2, which is involved in triglyceride biosynthesis (15). CGL2 is the most severe form of generalized lipodystrophy and is caused by mutations in *BSCL2* gene. The *BSCL2* gene, located on chromosome 11q13, encodes a transmembrane protein called seipin (16). Seipin has been postulated to have an important role in lipid droplet assembly, in adipocyte differentiation, and in lipid droplets fusion. Patients with CGL2 show an almost complete lack of both metabolically active and mechanical adipose tissues. These patients have an increased prevalence of cardiomyopathy and mild mental retardation compared to healthy individuals (4). In this study, we examined the clinical features, molecular characteristics, and modes of treatment of three Chinese patients with CGL from independent families in order to better understand the diagnosis, clinical treatment, and prognosis of the condition.

## METHODS

Three Chinese patients aged 2 to 6 months with generalized absence of subcutaneous adipose tissues and with a clinical suspicion of CGL were studied. They were all born to healthy and non-consanguineous parents. All three patients are from unrelated families, and they are residents of three provinces, namely, Guangdong province in southern China, Jiangxi and Hubei provinces in central China. Clinical data were collected by retrospective review of medical records. The clinical diagnosis of CGL was established by presence of muscular hypertrophy, hepatomegaly, insulin resistance, and hypertriglyceridemia, which are features of generalized lipodystrophy. Informed consent was obtained from the parents of all patients. The study was approved by the Institutional Review Board of Guangzhou Women and Children’s Medical Center. The mutational analysis of CGL gene was performed at Guangzhou Women and Children’s Medical Center from May 2012 to May 2015.

Blood glucose, cholesterol, and triglyceride levels were determined according to standard methods, using automated equipment. Serum insulin, follicle-stimulating hormone (FSH), and testosterone (T) levels were measured by chemiluminescence immunoassay (ADVIA Centaur XP Immunoassay Systems, Siemens, Erlangen, Germany). HbA1c was measured by immunoassay using the DCA Vantage Analyzer (Siemens Healthcare Diagnostics, Deerfield, IL, USA). Presence of hepatomegaly was assessed by abdominal ultrasound, and cardiac function was assessed using echocardiography and electrocardiography.

### Mutational Analysis

Genomic DNA was extracted from the peripheral leukocytes of the patients and their parents using a standard procedure. All exons and exon–intron splice junction regions of *AGPAT2* and *BSCL2* genes were amplified by PCR (Mastercycler Pro TM Gradient Thermal Cycler, Eppendorf, Hamburg, Germany). PCR products were purified and sent to BGI (Beijing, China) for direct DNA sequence analysis (DNA Analyzer3730, ABI, USA). Sequences were analyzed using Chromas software (V.2.01, Technelysium Pty Ltd, Tewantin QLD, Australia). Genetic variants were searched in the dbSNP, Clin Var, ExAC, and HGMD.

### Treatment and follow-up

Patients 1 and 3 were fed with low-fat breast milk at diagnosis and patient 2 was fed with milk powder containing medium-chain fatty acid. For patient 1 who had overt diabetes, insulin was administered subcutaneously in doses 2 U/kg daily for 1 month and decreased to 1 U/kg daily in the following two months. Clinical follow-up started with diagnosis at 1 month and then 2–6 months intervals, subsequently. Height, weight, and laboratory evaluation were measured at every visit. For patient 1, the data of self-monitoring blood glucose were also recorded.

## RESULTS

**Figure 1:**
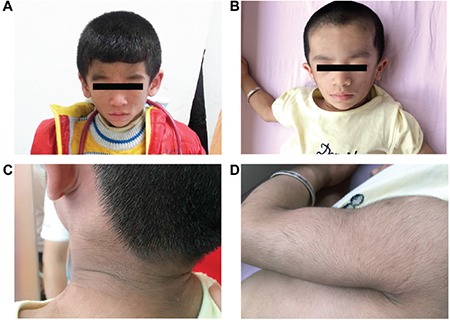
Clinical features of the three congenital generalized lipodystrophy patients indicate typical facial features with hollow cheeks and prominent masseters in patient 1 (A) and patient 3 (B); acanthosis nigricans (C), muscular hypertrophy and hirsutism (D) in patient 3.

The clinical and biochemical data on these patients are presented in Tables 1 and 2. Generalized lipodystrophy, acanthosis nigricans, muscular hypertrophy, hirsutism, hepatomegaly, and fatty liver were features in all three patients (Figure 1). All three patients had mild intellectual impairment with developmental language disorders.

Patient 1 was referred to our center at the age of 2 months for elevated glucose levels and poor weight gain. Physical examination was noteworthy for the generalized absence of subcutaneous adipose tissues; hypertrophy of all limb muscles; acanthosis nigricans in the neck; hirsutism in the neck, back and limbs; enlarged hands and feet. Additionally, he was noted to have emotional excitability and hyperactivity. The patient was diagnosed to have insulin resistance (fasting insulin 186 μIU/mL and fasting C-peptide 14.18 ng/mL) and diabetes mellitus [fasting blood glucose (FBG) 21.0 mmol/L] at the early age of 2 months. Laboratory examinations also indicated evidence of liver dysfunction [alanine aminotransferase (ALT) 219 U/L and aspartate aminotransferase (AST) 84 U/L] and dyslipidemia with markedly increased triglyceride levels and slightly decreased high-density lipoprotein (HDL) (22.17 mmol/L and 0.52 mmol/L, respectively). Additionally, ultrasonography revealed an enlarged liver with homogeneously increased echogenicity indicating steatosis. The boy was started on low-fat breast milk feedings, and insulin was administered subcutaneously in a dose of 2 U/kg daily. After one month, blood glucose was under control (FBG 3.9–8.3 mmol/L, postprandial blood glucose 5.0–15.0 mmol/L). At that time, the treatment was changed from insulin to glibenclamide. This fast reduction in insulin and switch of therapy to oral hypoglycemic drugs resulted in a rapid increase of glucose level. As a result, insulin treatment was resumed with a decreased dose of 1 U/kg daily. At the age of 6 months, he was weaned off insulin since his blood glucose was stable at 3.5–8.0 mmol/L. Feedings of low-fat breast milk led to a gradual decrease in serum lipid concentration (triglyceride 5.70 mmol/L). At the age of 1 year and 10 months, the boy returned to our center with a raised random blood glucose (17.2 mmol/L) and severe insulin resistance (insulin >300 μIU/mL and C-peptide 14.30 ng/mL).

Physical examination of patient 2 revealed a generalized and severe reduction of subcutaneous fat, prominent limb muscles, enlargement of the liver, and hirsutism in the neck, back, and limbs. Laboratory examination indicated that serum triglyceride level was raised (16.17 mmol/L). Her insulin and C-peptide levels were also high (59 μIU/mL and 5.05 ng/mL, respectively), but she had not developed diabetes until now. Echocardiography indicated an atrial septal defect, a left ventricular posterior wall thickness and a left ventricular outflow tract obstruction at 6 months of age. In addition to milk power containing medium-chain fatty acid, medical treatment with levocarnitine oral solution was given. After 1 month, her serum lipid concentration decreased dramatically (triglyceride 2.1 mmol/L). Now she is almost 3 years old and her triglyceride level is under control (Table 2).

The clinical and biochemical data of patient 3 are also given in Tables 1 and 2. In this patient, echocardiography revealed a patent foramen ovale. Ultrasonography demonstrated moderate hepatomegaly, fatty liver, and a small ovarian cyst (12 mm) when she was at an early age of 3 months. Three months later, the cyst was getting smaller (8 mm) and it disappeared at age 1 year.

## Genetic Analysis

Molecular alterations in the three patients are given in Table 3. No mutations were identified by sequencing all AGPAT2 exons and exon–intron junctions in our patients and their parents. Mutations in the *BSCL2* gene were found in all three patients (Table 3). All of these mutations have been previously reported. Our analysis showed that patient 1 and patient 2 carried the same compound heterozygous mutations, an insertion of a nucleotide, c.975insG in exon 7, resulting in a frameshift and truncated protein, G325fsX12 and a c.757G>T in exon 5, leading to a substitution of glutamic acid at codon 253 with a stop codon (Glu253X or E253X), respectively. Patient 3 had a homozygous c.545_546CGG trinucleotide insertion in exon 6.

## DISCUSSION

In this study, we examined the clinical and mutational features of three Chinese patients with CGL from unrelated families. Generalized lipodystrophy, acanthosis nigricans, muscular hypertrophy, hepatomegaly, insulin resistance, and hypertriglyceridemia were features present in all three patients. It is unusual to have diabetes mellitus early in life in patients with CGL. Though metabolic abnormalities of hyperinsulinemia and insulin resistance are evident early in life, overt diabetes generally develops during the pubertal years or adulthood (5,6,7,8,17). In a report by Van Maldergem et al (5), among 24 patients with BSCL2, 16 were diagnosed to have diabetes. However, the mean age of onset of clinical diabetes was 17.9 years. According to the report by Agarwal and his colleagues (6), patients with BSCL2 mutation had an onset of diabetes at a median age of 10 years. Recently, a nationwide study from Turkey showed that the mean age of onset of diabetes in these patients was 16.5 years (8). CGL patients who were diagnosed as insulin resistant in infancy and who developed diabetes mellitus at puberty have also been reported (7,17). Consistently, serum insulin levels of patient 2 and patient 3 in our study were high, but they had not developed diabetes so far. The unusual feature of our patient 1 was the development of diabetes mellitus at the very early age of 2 months. There are only two patient reports of overt diabetes mellitus described at ages 4 and 5 months (12,18). Our patient is the youngest reported CGL patient with diabetes mellitus so far.

Hypertrophic cardiomyopathy is reported in 20–25% of CGL patients and is a significant cause of morbidity from cardiac failure and of early mortality (9,10,11,12). However, this life-threatening complication is usually reported in older patients. Bjornstad et al (9) reported 6 patients of BSCL presenting with myocardial hypertrophy. Rheuban et al (10) described 4 other similar patients. According to their results, the average age for diagnosis of hypertrophic cardiomyopathy in CGL was 20 years. Similarly, Lupsa et al (19) reported 44 patients with lipodystrophy and pointed out that the average age of developing cardiac abnormalities in these patients was about 20 years. There are at least four patients with an early onset of hypertrophic cardiomyopathy who have been reported. Bhayana et al (11) described a young girl with CGL harboring a mutation in BSCL2, who was found to have myocardial hypertrophy from 6 months of age. In a 4-month-old Chinese boy, Friguls et al (12) reported severe cardiomyopathy with cardiac failure and systemic hypertension. Debray et al (14) reported a young boy presenting at age 1 month with severe hypertrophic cardiomyopathy. Miranda et al (13) also reported a 2-month-old boy harboring cardiomyopathy due to a homozygous missense mutation in BSCL2. In this present study, patient 2 was diagnosed with hypertrophy cadiomyopathy when she was aged 6 months. Echocardiography indicated atrial septal defect, an asymmetric left ventricular posterior wall thickness and a left ventricular outflow tract obstruction. The mechanism causing hypertrophic cardiomyopathy in patients with CGL is unclear. Theoretically, the severe insulin resistance observed in these patients may prompt cardiomyocyte hypertrophy by activating IGF-1 receptors which are largely expressed in the myocardial tissue and stimulate cell growth (10). Further studies are needed to elucidate the exact mechanism.

As mentioned before, dyslipidemia, observed in all three patients, was characterized by an increase in triglyceride levels and a decrease in HDL. TG levels decreased dramatically after exposure to a strict low-fat and low-sugar diet. This finding indicates that diet control is beneficial for CGL patients.

Four different CGL syndromes have been defined. Type 1 is associated with AGPAT2 mutations, type 2 with BSCL2 mutations, type 3 with CAV1 mutations, and type 4 with PTRF mutations. AGPAT2 mutations were found predominantly in patients of African ancestry which means that CGL1 is the major type of CGL in populations of African origin. Type 2 CGL has been reported in patients of various ethnicities including patients from Norway, United Kingdom, and Mediterranean countries, as well as Middle Eastern Arabs (5). Racial differences in the pathogenesis of CGL may exist, but the specific mechanisms leading to these differences need to be further elucidated. Gene mutation analysis was performed in our patients. We did not find any AGPAT2 mutation in these patients, but all of them had mutations in the *BSCL2* gene. Patient 1 and patient 2 had the same compound heterozygous mutations, one mutation was inherited from the mothers [c.757G>T (p.Glu253Ter)] and the other from the fathers [c.975insG (p.Gly325=fsX12)]. These two patients in our report had the same mutation of the same gene but revealed different clinical phenotypes. Patient 1 developed severe hypertriglyceridemia and diabetes mellitus at early infancy, while patient 2 had a much lower triglyceride level and no diabetes until now. Patient 2 had hypertrophic cardiomyopathy, which was absent in patient 1. These findings may suggest that the disease phenotype is not determined by the mutation alone and that other factors can contribute to the development of the clinical features in these patients. A homozygous mutation (c.545_546insCGG) of BSCL2 was found in patient 3. All of these mutations have been reported previously. Although the number of subjects we examined is small, these observations indicate that BSCL2 is a major causative gene for CGL in the Chinese people. According to previous reports, almost all Chinese CGL patients reported had mutations in BSCL2 (20,21,22,23), which is consistent with our results. The findings of this study may be helpful in expanding our knowledge of genotype-phenotype correlations in CGL patients.

## Figures and Tables

**Table 1:**
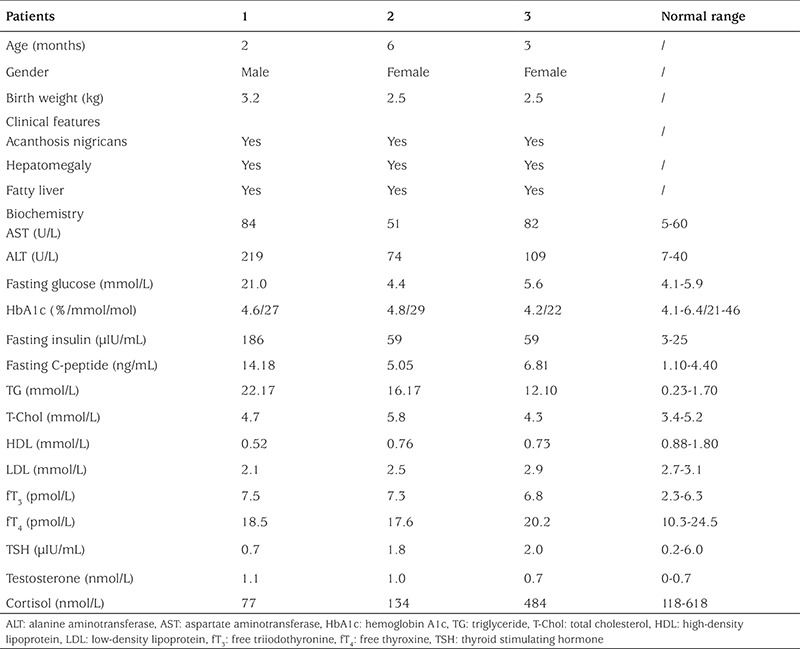
Clinical and biochemical data of the congenital generalized lipodystrophy patients at diagnosis

**Table 2:**
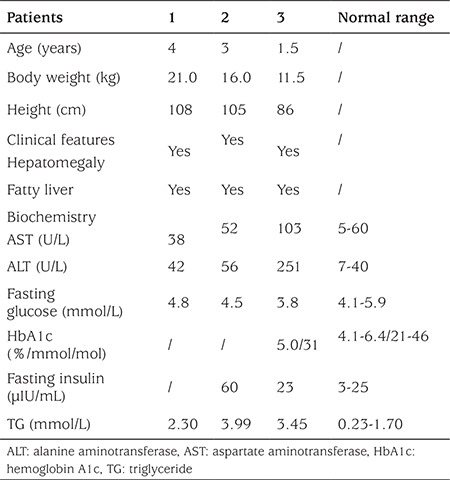
Clinical and biochemical data of the congenital generalized lipodystrophy patients at the most recent follow-up

**Table 3:**
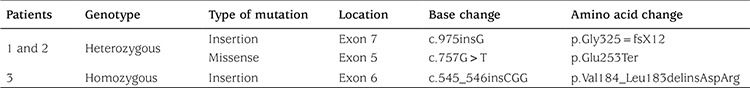
Molecular alterations in BSCL2 in the three patients
